# Novel Collagen-Based Emulsions Embedded with Palmarosa Essential Oil, and Chamomile and Calendula Tinctures, for Skin-Friendly Textile Materials

**DOI:** 10.3390/ma17153867

**Published:** 2024-08-05

**Authors:** Laura Chirilă, Miruna S. Stan, Sabina Olaru, Alina Popescu, Mihaela-Cristina Lite, Doina Toma, Ionela C. Voinea

**Affiliations:** 1 National Research and Development Institute for Textiles and Leather–INCDTP, Lucrețiu Pătrășcanu 16, 030508 Bucharest, Romania; laura.chirila@incdtp.ro (L.C.); sabina.olaru@incdtp.ro (S.O.); alina.popescu@incdtp.ro (A.P.); cristina.lite@incdtp.ro (M.-C.L.); doina.toma@incdtp.ro (D.T.); 2 Department of Biochemistry and Molecular Biology, Faculty of Biology, University of Bucharest, 91–95 Splaiul Independentei, 050095 Bucharest, Romania; ionela-cristina.voinea@bio.unibuc.ro

**Keywords:** palmarosa essential oils, plant tinctures, keratinocytes, biocompatibility

## Abstract

Skin-friendly textile materials were obtained by applying oil-in-water emulsions based on palmarosa essential oil, chamomile, and calendula tinctures onto cotton fabrics. Different formulations based on these bioactive principles incorporated in collagen as polymeric matrices were prepared and immobilized on a plain weave textile structure from 100% cotton. The functionalized textile materials were characterized in terms of physicochemical, mechanical, antibacterial, and biocompatibility points of view. The pH values of the prepared emulsions were in the range of 4.81–5.23 and showed no significant differences after 4 h of storage. Moreover, the addition of a higher quantity of active principles (palmarosa essential oil and plant tinctures) caused slightly lower values of acidic pH. The electrical conductivity of the obtained emulsions increased with the decrease in the oil phases in the system. The highest values were obtained for the emulsion developed with the smallest volume fraction of active principle—palmarosa essential oil and plant tinctures. The emulsion that contained the least amount of collagen and the highest number of active principles exhibited the lowest stability. The textile materials treated with synthesized emulsions exerted antibacterial effects against *S. aureus* and *E. coli* strains and did not affect keratinocyte growth, spreading, and organization, highlighting the biocompatibility of these developed skin-friendly textiles.

## 1. Introduction

The increase in skin sensitivity to different allergens, such as chemical substances in cosmetics, pollution, insects, etc. [1,2,3], is corroborated by the occurrence of certain domestic accidents, such as first-degree burns, cuts, or scratches (especially at children), culminating in the necessity to study the skin–textile interaction [4] in order to develop skin-friendly textile materials. Besides the choice of the textile material, from which cotton is, by far, the most preferred [5], it is important to select the appropriate manufacturing processes, as textile finishing is the determinant step for conferring the desired properties to the final product [6]. The use of natural finishes constitutes an appealing option for preventing skin irritation caused by the presence of more chemical compounds. Natural finishes consist of plant extracts [7], biopolymers [8], green synthesized nanoparticles [9], essential oils [10], etc., and their advantages lie in their biodegradability and renewability, which goes along with their biocompatibility and nontoxic character [11]. The exploited properties of these finishers include not only antimicrobial properties [12] but also healing and regenerating properties [13], together with the relaxing and calming effects [14], which are derived from their phyto-constituents, such as flavonoids, phenolic compounds, terpenes, saponins, etc., which possess antioxidant, anti-inflammatory, antiseptic, and wound-healing properties [13,15,16].

Thakker reported the use of *Sapindus mukorossi* and *Acacia concinna* extracts in cotton fabrics processing and observed the presence of saponins such as oleanolic acid, diosgenin, soyasaponin, and sarsasapogenin in both the herbal extract powders and the cotton fabrics treated with herbs, which have the potential to confer healing properties [17]. A key aspect of wound or burn healing is the antimicrobial properties conferred on the support material. Ketema and Worku studied the effect of stinging nettle (*Urtica dioica* L.) plant leaf extract as an antibacterial finishing and found that high percentages of bacteria reduction are obtained after applying the pad–dry–cure treatment on cotton fabrics [18]. 

Another method to produce antimicrobial finishing using plant extracts is to synthesize metal nanoparticles, such as silver nanoparticles, by reducing the silver salts using the phytoconstituents present in the extract and applying the resulting dispersion on the textile fabrics [19]. Different systems have been reported for embedding natural compounds into textile fibers, such as agar systems [20], chitosan [21,22,23], collagen [23,24], and microcapsules [22,25,26]. 

Emulsion-based systems are widely used for essential oil blending [20,27]. In 2021, Danila et al. investigated the potential skincare properties of the lavender essential oil-based emulsion provided to textile fabrics [28], and, in 2023, Rosu et al. obtained aromatherapeutic and antibacterial cotton materials by the treatment of textiles with emulsions containing peppermint essential oil [29].

In order to increase biocompatibility, Berechet et al. reported the use of collagen hydrolysate-based nanofibers loaded with thyme or oregano essential oils for manufacturing wound dressings [30]. Similarly, palmarosa essential oil microcapsules applied on cotton knits were shown to carry antimicrobial properties in a study conducted by Kudligi et al. [31]. 

Palmarosa essential oil has been intensively used in aromatherapy and it has valuable characteristics in order to be used as an eco-friendly finishing agent on textiles to impart better functional properties. It is known for its antimicrobial, regenerating, and antioxidant activities [32]. Chamomile and calendula are widely spread medicinal herbs that possess not only anti-inflammatory and antioxidant properties, but also relaxing and calming properties, which were considered in order to produce a finishing system that confers antimicrobial properties as much as healing and regenerating properties.

The use of multifunctional coatings is widely recognized as a top strategy in combating the proliferation of pathogens. These coatings integrate diverse antimicrobial approaches, serving as multiple lines of defense to mitigate the inherent limitations associated with individual strategies.

Our study aimed to obtain skin-friendly textile materials by applying emulsions containing palmarosa essential oil, chamomile, and calendula tinctures, which can serve as experimental models for developing skin-friendly textiles designed for different topical applications (i.e., patches or bandages). The prepared oil in water emulsions (O/W) differ from each other by the concentration of the embedding agent (collagen) and the concentration of the selected bioactive compounds. For all six variants of emulsions, the ratio between the two selected tinctures was kept constant. The developed emulsions were studied by means of physico-chemical parameters (pH, conductivity, viscosity, creaming index). The fabrics treated with the synthesized emulsions were tested for their antibacterial efficiency and biocompatibility on human keratinocytes. 

Due to the well-known healing and regenerating properties of palmarosa essential oil and plant tinctures incorporated in the emulsions, the developed textile materials offer an alternative with a high therapeutic potential for a wide range of skin pathologies, considering both the multiple functionalities and the numerous biopharmacological advantages. Co-release of antimicrobial compounds with different mechanisms offers a double advantage because it simultaneously induces a reduced microbial resistance with a selected, synergistic antimicrobial action. The significant aspect of our work involves the use of a reduced amount of synthetic emulsifier Tween 80, which decreases surface tension, and the utilization of a natural, eco-friendly, and biodegradable polymer. Furthermore, calendula and chamomile tinctures are readily available and cost-effective medicinal plant products with well-established antimicrobial, anti-inflammatory, antioxidant, healing, and regenerating properties. These properties were taken into consideration for producing bioactive polymeric systems with diverse functions. Based on our knowledge and scanning the specialized literature, palmarosa essential oil is being used synergistically with calendula and chamomile plant tinctures for the first time.

## 2. Materials and Methods

### 2.1. Materials

Verisol^®^-type collagen peptides were purchased from Zenith, Neamț, Romania, Tween 80 was supplied by Sigma Aldrich, Darmstadt, Germany, and glycerol was purchased from Honeywell, Charlotte, NC, USA. Palmarosa essential oil—PEO (*Cymbopogon martinii*) was purchased from Ellemental^®^, Oradea, Romania, calendula tincture (1:5 hydroalcoholic extract of *Calendula officinalis*) and chamomile tincture (ethanolic extract 70% *v*/*v* of *Matricaria chamomilla*) from Dacia Plant, Brașov, Romania, were used as the dispersed phase. NaOH, Na_2_CO_3_, and Na_3_PO_4_ were supplied by Consors SRL, Bucharest, Romania, and Kemapon PC (nonionic wetting agent and detergent based on fatty alcohol polyglycol ether), Kemapol SR 40 Liq (dispersing and sequestering agent of Ca-Mg ions), and Kemaxil Liq H_2_O_2_ (stabilizer with dispersing and sequestering properties based on disodium salt of aminopolycarboxilic acid) were supplied by Kem Color S.p.a, Torino, Italy. Citric acid was purchased from Fluka™ Chemie GmbH, Buchs, Switzerland. 

For the development of skin-friendly textiles, 100% raw cotton fabric with 196 g/m^2^ was used, which is a proper weight for this kind of application. The cotton fabric has the following characteristics: warp and weft yarn count: Nm 60/2, warp density: 30 ends/cm, and weft density: 23 picks/cm.

### 2.2. Formulation of Oil-in-Water (O/W) Emulsions

Collagen-based emulsions loaded with palmarosa essential oil and plant tinctures were prepared by an emulsification procedure in accordance with the method described in one of our previous studies [12], with small modifications. Firstly, solutions of 5% collagen and 30% Tween 80 were prepared, and then the emulsions were obtained according to the recipes presented in Table 1, being mixed under vigorous magnetic stirring for 10 min at each stage. After complete homogenization of the resulting polymeric systems, palmarosa essential oil, calendula, and chamomile tinctures were dropwise separately added, maintaining the magnetic stirring for 10 min at each stage. All the steps were carried out at a room temperature of 22 °C.

### 2.3. Immobilization of Bioactive Polymeric Systems on Fabrics 

To ensure proper hydrophilicity of the textile fabrics, and to facilitate the functionalization process, two successive steps of preliminary preparation were applied: hot alkaline treatment and bleaching. The textile materials were first subjected to a hot alkaline treatment at 95 °C for 90 min. The treatment bath consisted of 4 mL/L NaOH (38° Be), 3 g/L Na_2_CO_3_, 3 g/L Na_3_PO_4_, 2 g/L Kemapon PC, and 1.5 g/L Sequion. Following this incubation, the fabrics were washed repeatedly at 80 °C, 60 °C, 40 °C, and at room temperature for 10 min. Afterward, the fabrics were subjected to a bleaching operation at 98 °C for 60 min using the following recipe: 20 g/L H_2_O_2_, 4 g/L NaOH (38° Be), 2 g/L Kemaxil, and 1 g/L Kemapon PC. Next, the fabrics were exposed to a bleaching process at 98 °C for 60 min with the following recipe: 20 g/L H_2_O_2_, 4 g/L NaOH (38° Be), 2 g/L Kemaxil, and 1 g/L Kemapon PC. Afterward, the fabrics were rinsed multiple times under the following conditions: warm rinses for 10 min each at 90 °C, 60 °C, and 40 °C, and, finally, a cold wash. In order to adjust the pH of the fabrics, the textile materials were immersed in a solution containing 4 g/L citric acid for 20 min, followed by rinsing with hot and cold water. After these treatments, the fabrics were squeezed and dried freely at room temperature.

The functionalization of textile materials with the six variants of developed emulsions (Table 1) was performed by the padding method. A simple laboratory-scale padder BVHP 2 (Roaches, West Yorkshire, UK) with two rollers was used in the following conditions: 2 passes, 1 m/min, with a pressure of 0.74 bar. The fabrics were padded twice with the emulsions until they are until a wet pick-up rate of approximately 90% was reached when the fabrics were completely saturated with emulsions. The drying operation of the textile materials was carried out at a temperature of 50 °C for 3 min.

### 2.4. Emulsions’ Characterization 

#### 2.4.1. pH Measurements

The pH value of emulsions was directly measured at 22 ± 1 °C using a HANNA portable pH meter immersed in the prepared undiluted emulsions. 

#### 2.4.2. Conductometric Analysis

The conductivity of analyzed emulsions was measured directly at 22 °C using the Consort conductometer, model C1020 WTW (TARA), with the SP10T electrode. The conductivity values represent the average of 3 successive readings made on the same sample.

#### 2.4.3. Creaming Index (CI)

To investigate the potential for phase separation, the freshly prepared emulsions were immediately transferred into 10 mL cylinders. The creaming index (CI%) was calculated using Equation (1):

CI% = 100 × (Hc/H_0_), (1)

H_0_ represents the total height of the emulsion layer, while H_C_ stands for the height of the cream layer. The measurements for H_C_ and H_0_ were conducted in triplicate, immediately after preparation and again after 8, 24, 48, and 72 h of storage (22 ± 1 °C).

#### 2.4.4. Viscosity Determination

The viscosity measurements of the resulting emulsions were conducted using the DV2T Brookfield AMETEK viscometer (AMETEK Brookfield, Middleboro, MA, USA) with the measurements being taken in triplicate.

### 2.5. Characterization of the Functionalized Textile Materials

#### 2.5.1. Physicochemical and Mechanical Characteristics

The treated fabrics were characterized based on their main physicochemical and mechanical properties. These properties include mass per unit area [33], air permeability [34], and water vapor permeability [35] (SR 9005: 1979). Hydrophilicity was assessed using Romanian Standard SR 12751-89 [36], which involves determining fabric wettability using a drop method.

#### 2.5.2. Assessment of Antibacterial Activity

The textile materials’ antibacterial activity was assessed using the Agar diffusion plate test according to the ISO 20645:2004 standard [37] by using cultures in a liquid medium replicated at 24 h of ATCC 6538 *Staphylococcus aureus* (Gram-positive) and ATCC 11229 *Escherichia coli* (Gram-negative) test strains. The samples were cut into 20 mm diameter circles and placed in the middle of Petri dishes containing the bacteria cultures. The culture medium was poured into two layers in Petri plates. The lower agar layer consisted of 10 mL of nutrient agar, while the upper layer consisted of 5 ± 1 mL of agar inoculated with bacteria. Specifically, 1 mL of bacteria working solution with a concentration of 1.5 × 10^8^ colony forming units (CFU) was added per 150 mL of agar. After 48 h of incubation at 37 ± 1 °C, the samples were assessed for the absence or presence of bacterial growth in the contact zone between the agar and the sample, as well as for the potential appearance of the inhibition zone (H), which was calculated using the following formula:

H = (D − d)/2(2)

where D—the total diameter of the specimen and inhibition zone (measured in mm) and d—the diameter of the specimen (measured in mm).

In accordance with the standardized procedure, the diameter of the inhibition zone was calculated in millimeters, and the extent of bacterial growth was assessed within the nutrient medium surrounding the specimen. Based on the ISO 20645 method [37], the antimicrobial efficacy of the analyzed samples was categorized as “good”, “limited”, or “insufficient”.

#### 2.5.3. Measurement of Keratinocytes’ Response to Fabrics’ Exposure

Biocompatibility testing of fabrics impregnated with bioactive polymeric systems involved culturing human keratinocytes (HaCaT cell line) in Dulbecco’s Modified Eagle Medium (DMEM, Sigma-Aldrich, St. Louis, MO, USA) supplemented with 10% fetal bovine serum (FBS, Gibco, Grand Island, NY, USA) at 37 °C in a 5% CO_2_ humid atmosphere, following a methodology previously described by Chirila et al. [12]. 

Two types of exposure were performed to assess the biocompatibility of emulsion-impregnated fabrics. The first one included the incubation of HaCaT cells with the extracts obtained from each sample. For the preparation of these fabric extracts, we followed the steps described by Fanizza et al. [38]. Small pieces (0.3 cm × 0.3 cm) of each fabric sample were sterilized under UV light for 72 h and incubated in 1 mL of DMEM with FBS in test tubes for 24 h at a speed of 240 rpm/min. The cells added onto 96-well plates at a density of 3 × 10^4^ cells/well and left to adhere overnight were incubated with 100 µL of fabric extracts (conditioned media) for 24 h. The second approach involved the direct contact of cells with fabrics. The cells were added to 24-well plates at a density of 10^5^ cells/cm^2^, and the sterilized textile samples (0.3 cm × 0.3 cm) were placed on the adhered cells for 24 h. 

The viability of cells was measured by MTT [3-(4,5-dimethithiazol-2-yl)-2,5-diphenyltetrazolium)] assay, which involves the reduction of MTT to purple formazan. Nitric oxide (NO) production was determined using the Griess colorimetric method, where the optical density of the medium mixed with Griess reagent was read at 550 nm [12]. Lactate dehydrogenase release was measured to check the integrity membrane after incubation with fabrics. A total of 50 µL of cell culture supernatant was incubated for 30 min at room temperature with a 50 µL mix of dye and catalyst provided by a Cytotoxicity detection kit (Roche, Mannheim, Germany), the absorbance being read at 490 nm with FlexStation 3 (Molecular Devices, San Jose, CA, USA). Statistical analysis was assessed by a one-way analysis of variance (ANOVA) test followed by the Bonferroni post-test, with a value of *p* < 0.05 being set as significant.

The LIVE/DEAD^TM^ assay kit (Molecular Probes, Life Technologies Corporation, Johannesburg, South Africa) was used in accordance with the guidelines of the manufacturer to visualize the distribution of viable and non-viable keratinocytes after 24 h of incubation with fabrics’ extracts. Images were taken on Olympus IX71 (Olympus, Tokyo, Japan).

Cells exposed in direct contact with the fabrics were fixed for 20 min with 4% paraformaldehyde and permeabilized 0.1% Triton X-100 in PBS for 45 min. The Actin cytoskeleton was labeled with phalloidin-iFluor 555 reagent (Abcam, Cambridge, UK) for 40 min in the dark. Representative images were captured on an Olympus IX71 with a 20× objective.

## 3. Results and Discussion

### 3.1. Physicochemical Analysis of Emulsions

It is essential to carefully select the surfactant and determine its concentration when preparing emulsions. Surfactant concentrations are typically chosen to be higher than the critical micellar concentration (CMC), which is the point at which micelles are forming. Below this concentration, the surfactant exists in its monomeric form. For instance, in aqueous solutions, the CMC for Tween 80 is 0.014 g/L [39,40]. Our study explores the interaction of a surfactant (at a constant concentration) with one essential oil, plant tinctures, and biopolymer, as well as their solubilization method in micelles and the capture of monomers. Van der Waals forces are responsible for the interaction process of the surfactant with the components of the system (biopolymer/oily or alcoholic extracts). For this reason, the concentration of surfactant was kept constant, and the proportion of biopolymer was changed, following the way of its incorporation (clamping) in micelles or monomers, until the phenomenon of saturation, considering the maximum of 50 mL volume for surfactant–biopolymer interaction. Glycerol can be used as the hydrophilic part of nonionic surfactants, changing the system’s hydrophilic–hydrophobic balance (HLB). The HLB value represents the ratio of the structure’s weight percentages of hydrophilic and hydrophobic groups. Under controlled conditions, a constant amount of Tween 80, a surfactant with a long hydrophobic chain, was added, while glycerol solely increased the system’s hydrophilic weight. The concentrations of palmarosa essential oil and plant tinctures varied, keeping constant the amount of surfactant (Tween 80) because we followed their solubilization in micelles. As an effect, the mode of interaction between surfactant and other components (biopolymer, essential oil, and plant tinctures) was monitored, which could only be achieved by changing the concentration ratios, as shown in Table 1.

The pH and electrical conductivity values recorded for the synthesized emulsions are presented in Table 2.

After analyzing the pH values of the emulsions immediately after their synthesis, it is observed that they fall within the 4.81–5.23 range. Additionally, it can be noticed that higher quantities of active principles (such as palmarosa essential oil and plant tinctures) caused slightly lower acidic pH values. Furthermore, there was no significant difference in pH values after 4 h of storage. It is important to mention that the pH of the skin surface is typically acidic, ranging between 4 and 6 [41]. The physiological function of an acidic skin surface pH is to provide protection against microorganisms. The developed emulsions have pH values between 4.81 and 5.23, making them suitable for topical applications. 

Conductivity measurements were carried out in order to study the conductometric behavior of the freshly prepared multiple emulsions and also to evaluate the stability after preparation (0 h) and after 4 h of storage (22 ± 1 °C). It should be noted that any significant decrease in conductivity over time indicates weak stability of size and implies the loss of integrity, eventually leading to coalescence. The electrical conductivity of the emulsions ranged from 1.21 to 1.47. The highest values were observed for the emulsion containing the smallest volume fraction of the active principle, palmarosa essential oil, and plant tinctures (e.g., R4 sample: φ = 0.02 mL active principle/mL emulsion). This can be explained by the poor conductive characteristics of the essential oil. Analysis of electrical conductivity showed reduced values for all emulsions with the same volume fraction of the active principle (R1–R3 samples: φ = 0.06 mL active principle/mL emulsion). The conductivity values decreased as the water content decreased. Additionally, conductivity values did not show significant changes after 4 h of storage.

### 3.2. Stability of the Emulsions

**Figure 1:**
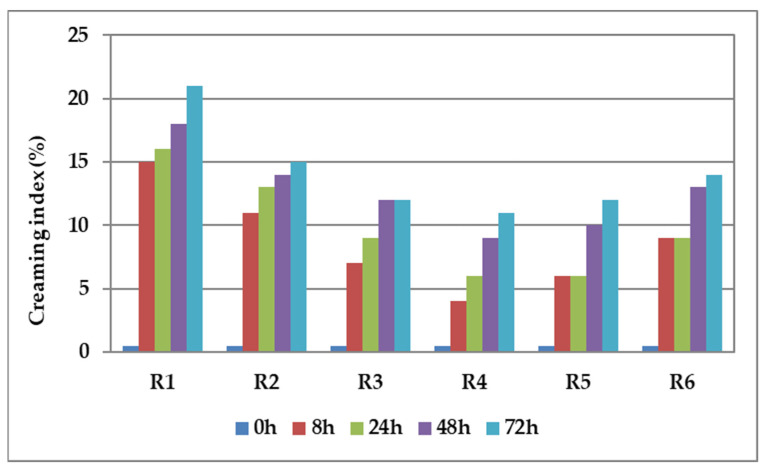
Variation in time of the creaming index values for the emulsions containing palmarosa essential oil and plant tinctures.

For practical applications, it is essential to ensure the long-term stability of the emulsions. At 22 °C, the stability of the emulsion was assessed by examining the creaming index over storage periods of 8, 24, 48, and 72 h. The variations in the calculated creaming index values are graphically depicted in Figure 1. 

An emulsion is a dynamic system involving two liquids that typically do not mix. One of the liquids is dispersed as droplets throughout the other, creating a unique and versatile mixture with various practical applications [42]. The stability of an emulsion depends on the type and quantity of surfactants present. These surfactants help the emulsion remain stable by creating a film around the water droplets at the water–oil interfaces [43]. Emulsions exhibit inherent thermodynamic instability, leading to the separation of oil and water phases. This phenomenon is driven by processes such as gravitational separation flocculation, sedimentation, coalescence, Ostwald ripening, creaming, and phase inversion [43]. Among these phenomena, creaming and sedimentation occur due to gravitational forces, causing droplets to cluster, grow in size, and accumulate at the top of the emulsion. In biphasic systems, creaming is a natural phenomenon caused by gravity indicating an emulsion’s destabilization. Creaming usually precedes coalescence and ultimately leads to phase separation. The creaming index (CI) is a parameter that indirectly indicates the agglomeration of emulsion droplets and serves as a valuable metric for assessing emulsion stability. Emulsions with a low creaming index exhibit favorable creaming behavior, indicating strong stability. A creaming index between 0 and 20% suggests there is minimal serum separation and indicates a higher level of emulsion stability [44]. 

The fresh samples showed good stability against coalescence, sedimentation, and creaming. After 8 h of storage, even in conditions of using a higher content of active principle, there was no emulsion creaming process. After a 72 h storage period, there was a slightly visible sedimentation, which resulted in the formation of a layer of cream at the bottom of the glass cylinders due to the density disparity of the two phases. The findings indicate that emulsion R4 demonstrated the highest stability (which contains the higher content of collagen and lower content of bioactive principles, with φ = 0.02 mL active principle/mL emulsion) while emulsion R1 (which contains the least amount of collagen, with φ = 0.06 mL active principle/mL emulsion) exhibited lower stability. 

The inclusion of collagen is indispensable in stabilizing emulsions with favorable foaming properties. It achieves this by diminishing the surface tension at the air–liquid interface through an increase in the viscosity of the aqueous phase. Each emulsion contains an equivalent quantity of Tween 80, a component essential for ensuring the fine dispersion of oil particles by decreasing the surface tension at the oil—water interface [43,45].

### 3.3. Viscosity of the Emulsions

The main viscosity indices obtained for the synthesized emulsions are shown in Table 3.

When performing a comparative analysis of the viscosity values, it can be seen that the experimental variant R6 has the highest value. (241.22 cP) in which the highest volume fraction of collagen and the smallest volume fraction of water were used, while the variant R1 exhibits the lowest viscosity. Also, it was observed that the same level of collagen volume fraction (R3–R6 variants) have different viscosities, the sample R5 being characterized by the smallest viscosity. 

It can also be observed that the viscosity of the developed emulsions remains unaffected by the volume fraction of the active principle. Even when the highest amount of active principle (R6: φ = 0.08 mL active principle/mL emulsion) is used, the viscosity values obtained are comparable to those obtained with smaller volume fractions (R4: φ = 0.02 mL active principle/mL emulsion, R1–R3: φ = 0.06 mL active principle/mL emulsion, R5: φ = 0.04 mL essential oil/mL emulsion). The shear stress (SS) reaches its highest value in the R6 variant, which is obtained by using the highest volume fraction of chitosan and the smallest volume fraction of water.

### 3.4. Physicochemical and Mechanical Characteristics of Cotton Treated Samples

The obtained values for the main physicochemical and mechanical characteristics of functionalized textile materials are presented in Table 4.

When considering the main mechanical properties of the treated textile materials, the results indicate that the applied functionalized treatments resulted in fabric shrinkage, resulting in a slight increase in mass for all analyzed samples. Furthermore, all emulsion-treated samples demonstrated reduced water and air permeability in comparison to the untreated sample. This behavior can be attributed to the components of the immobilized emulsion, in the form of a semi-permeable film, which would fill the gaps between fabric yarns, restricting airflow through the treated fabrics. The functionalized cotton fabrics show slightly lower hydrophilicity values, with a wettability range of 1.20–1.70 s. The R6 sample displays the most significant decrease in hydrophilicity, correlating with a reduced air permeability for this sample. Cotton fibers exhibited better hydrophilic properties since their surface has more bonding sites for water molecules than other natural or synthetic fibers. Additionally, when the polymeric systems were absorbed by textile materials, the fibers swelled up and the size of air spaces narrowed down, slowing down the diffusion process. 

### 3.5. Antibacterial Activity

**Figure 2:**
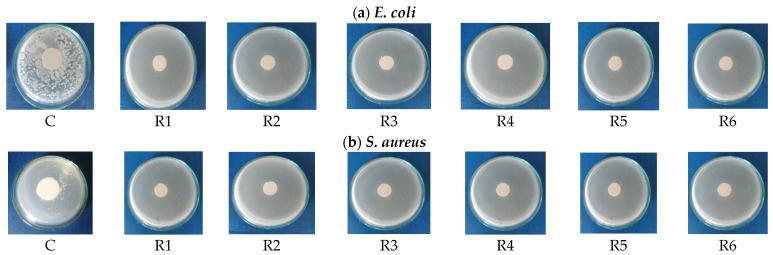
Representative images of Petri dishes revealing the antibacterial effect against (**a**) *E. coli* and (**b**) *S. aureus* strains, after 24 h in the presence of emulsion-treated fabrics.

Images of Petri plates after 24 h of incubation of cotton-treated fabrics with *E. coli* or *S. aureus* strains can be observed in Figure 2, and the assessment of antibacterial activity is presented in Table 5.

The efficacy of the emulsion-treated samples in demonstrating antibacterial activity was assessed by measuring the diameter of the inhibition zone. Analysis of the data reveals that the emulsions containing palmarosa essential oil and plant tinctures exhibit notable antibacterial activity against both *E. coli* and *S. aureus*. In all cases, the absence of bacterial strain growth throughout the culture medium signifies a notable antibacterial effect. Conversely, the untreated cotton sample displayed no antibacterial action against the two test strains, as evidenced by their significant development.

### 3.6. Biocompatibility Assessment on Human Keratinocytes

**Figure 3:**
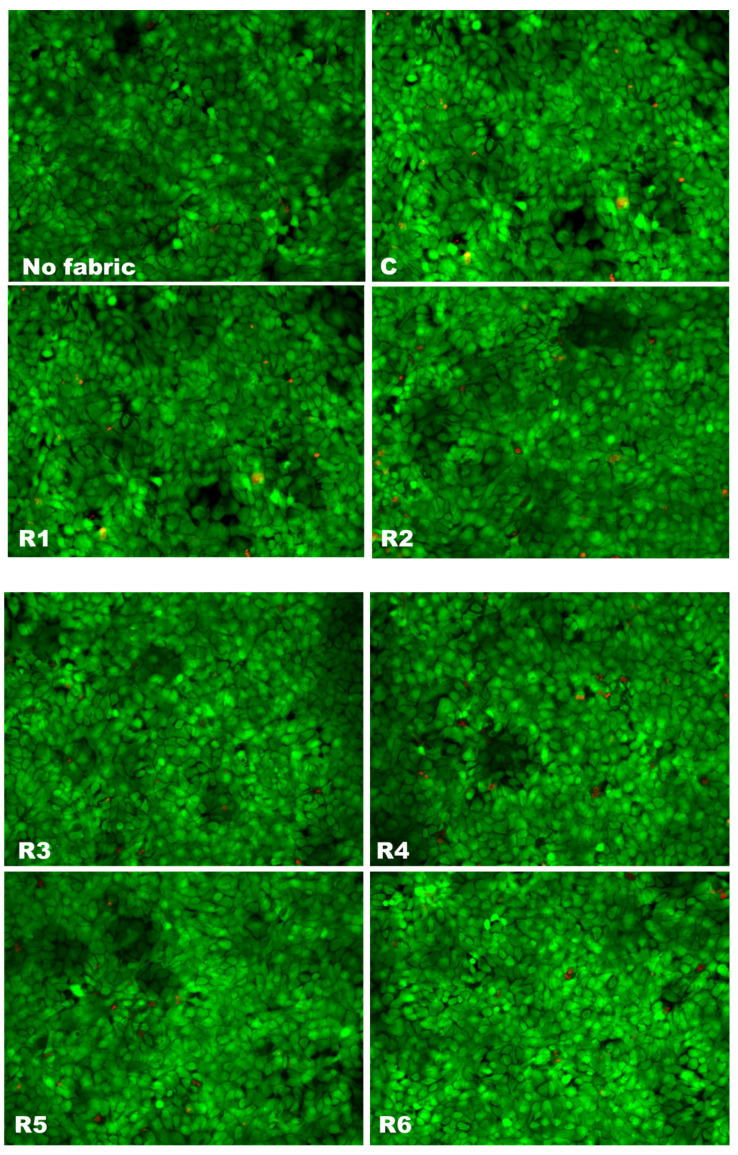
Fluorescence images of Live and Dead staining of human keratinocytes (HaCaT cells) grown for 24 h in the presence of extracts from emulsion-treated fabrics (R1–R6) or untreated fabrics (C). Live cells are shown in green (after staining with calcein AM solution) and dead cells are presented in red (labeled with propidium iodide solution). In parallel, cells without any fabrics were analyzed. All images were obtained with 20× objective.

**Figure 4:**
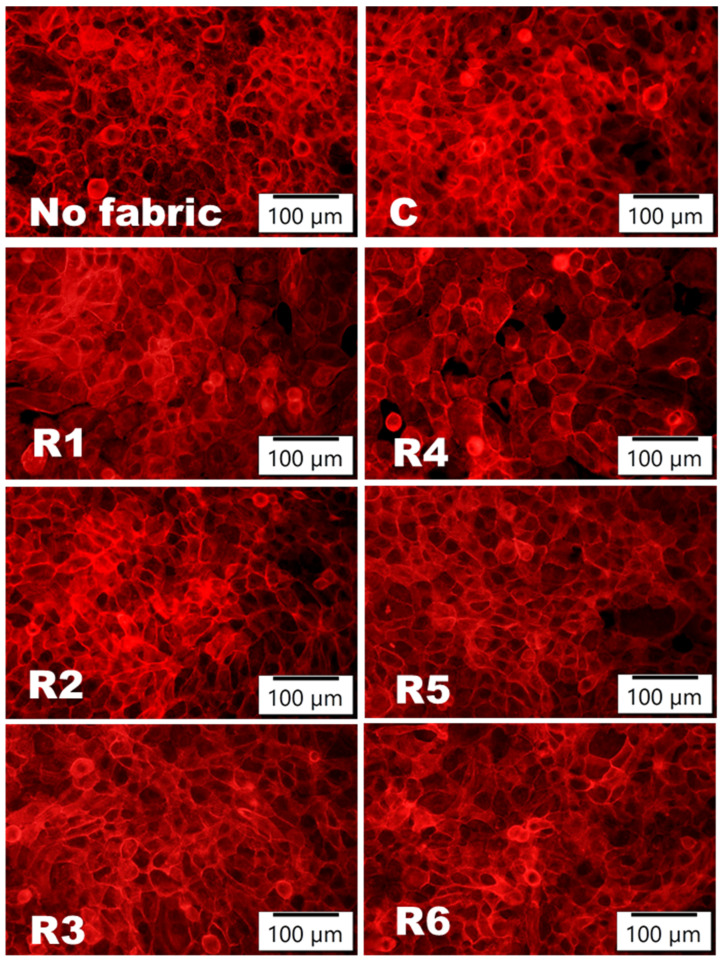
Organization of actin filaments (labeled in red with phalloidin-iFluor 555 reagent) in human keratinocytes (HaCaT cells) after 24 h of incubation in direct contact with emulsion-treated fabrics (R1–R6) or untreated fabrics (C). In parallel, cells without any fabrics were analyzed.

**Figure 5:**
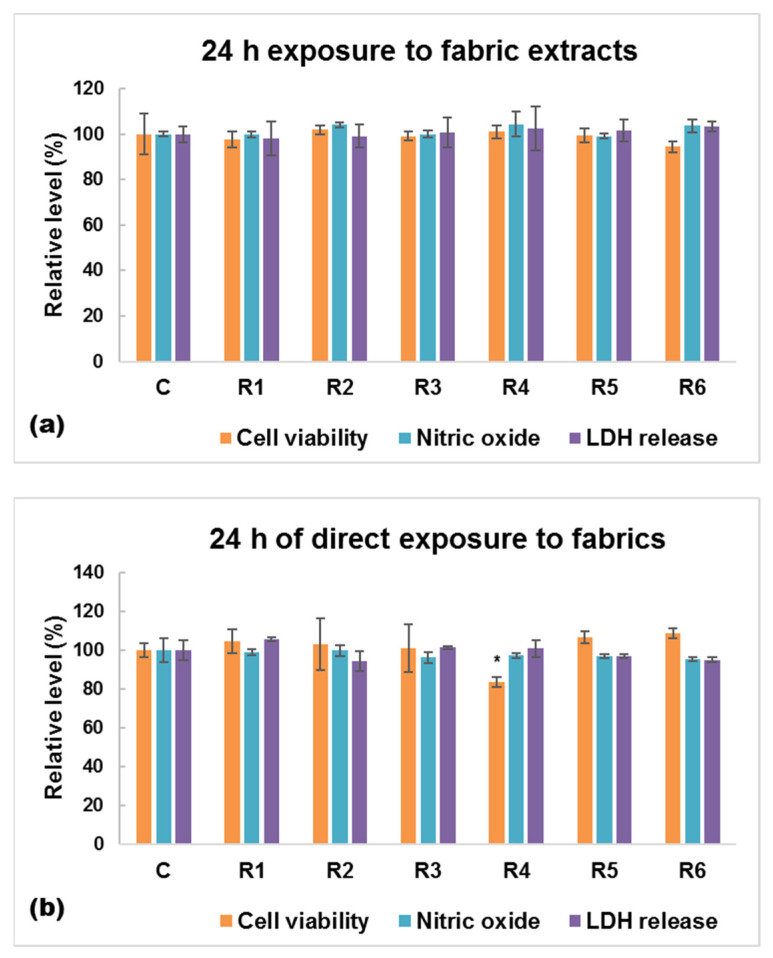
Biocompatibility in terms of cell viability, nitric oxide level, and lactate (LDH) dehydrogenase evaluated on human keratinocytes (HaCaT cells) after 24 h of growth: (**a**) in the presence of fabrics extracts, and (**b**) in direct contact with the emulsion-treated fabrics. Data are calculated as mean ± standard deviation (*n* = 3) and normalized to HaCaT cells grown: (**a**) in the presence of extracts from untreated fabric (C—control), (**b**) in direct contact with untreated fabric (C). No significance was obtained after statistical analysis was performed between fabric extracts. * *p* < 0.5 compared to untreated fabrics (C).

In order to develop skin-friendly textiles, human toxicity and, in particular, skin sensitization should be the primary assessment item. Therefore, the in vitro cytotoxic effects of the functionalized textile materials obtained within this study were assessed on human keratinocytes and the results are presented in Figure 3, Figure 4 and Figure 5. First of all, it is important to state that the weave fabric made from 100% cotton without any emulsion treatment (C sample) did not alter the cell viability or membrane integrity, regardless of the condition of exposure (Figure 3 and Figure 4), confirming the biocompatibility of this type of textile. The fluorescence images of live and dead cell staining (Figure 3) confirmed the absence of cell toxicity after 24 h of exposure to the extracts obtained from untreated and emulsion-treated samples. The number of dead cells (stained in red) remained very low per visual field for each tested sample, exactly like in the wells grown in the absence of any textile.

Moreover, the staining of the actin cytoskeleton, represented in Figure 4, allowed the visualization of keratinocytes’ organization in the case of direct contact with emulsion-treated fabrics. It can be observed that the 24 h contact with these samples did not affect the healthy distribution of actin filaments and the good spreading of keratinocytes on the culture surface. Therefore, the developed emulsions are very biocompatible with the human keratinocytes’ growth and organization.

When the human cells were exposed to conditioned media represented by fabric extracts, no significant changes in terms of cell viability, inflammatory response (NO release), and cell membrane permeabilization (LDH leakage) were recorded between control and cotton fabrics impregnated with essential oils (Figure 5a). Our data showed a slight difference between the tested samples only after direct exposure of the cells to these modified fabrics (Figure 5b). There was no evidence that the collagen concentration would have any contribution to increasing the biocompatibility of these new skin-friendly materials, but our findings showed that using a lower content of essential oils led to the obtaining of textiles with reduced biocompatibility. Therefore, sample R4 induced a 16% decrease in cell viability compared to untreated fabrics after a 24 h direct contact. 

The lack of an inflammatory response can be explained by the well-known antioxidant and anti-inflammatory abilities of essential oils. Several studies reported that calendula, chamomile, and palmarosa have the capacity to inhibit the production of nitric oxide and to reduce the cytokine levels of TNF-α, IL1, IL6, IL-8, and IL-12 [46,47].

To the best of our knowledge, the cytotoxic effects of textiles impregnated with chamomile, calendula, and palmarosa oils on skin cells have not been reported so far, but there are few studies reporting interesting biological effects exhibited by other essential oils. For example, clove essential oil was used by Huerta et al. for skin regeneration applications. Their cellulose nanofiber hydrogels loaded with clove oil displayed great biocompatibility with human gingival fibroblasts, making them a promising candidate for wound dressings [48].

In a previous study, Ngampunwetchakul et al. showed that ginger essential oil can be successfully used to reduce toxicity of semi-solid polyvinyl alcohol (PVA)-based hydrogels designed for wound treatment [44]. The ginger oil-loaded hydrogels proved great biocompatibility on NCTC clone 929 and NHDF cell lines [49].

Also, Singh et al. designed hydrogel membranes loaded with thyme essential oil with no toxicity and excellent biocompatibility on HEK293 cells [50].

## 4. Conclusions

Six emulsions containing palmarosa essential oil, chamomile, and calendula tinctures were prepared and then immobilized onto 100% cotton fabrics using the padding method. The developed emulsions were investigated to explore their physical, chemical, and biological properties for creating skin-friendly textile materials. The functionalization treatments had minimal influence on the main physicochemical and mechanical characteristics of cotton fabrics. Additionally, the textile materials treated with the developed emulsions exhibited antibacterial activity against *S. aureus* and *E. coli*. Our findings indicated that cotton fabrics treated with these emulsions had high biocompatibility with human keratinocytes. These overall results suggest that the cotton fabrics impregnated with emulsions based on collagen–palmarosa essential oil-plant tinctures can be employed as adequate candidates with the potential of being used in skin care products with single use, such as patches and bandages for wound healing and first-degree burns treatment.

## Figures and Tables

**Table 1:** Emulsions’ compositions.

Code	Collagen (mL)	Tween 80 (mL)	Glycerol (mL)	Distilled Water (mL)	Palmarosa Essential Oil (mL)	Calendula Tincture (mL)	Chamomile Tincture (mL)
R1	30	1.67	9	53.33	3	1.5	1.5
R2	40	1.67	9	43.33	3	1.5	1.5
R3	50	1.67	9	33.33	3	1.5	1.5
R4	50	1.67	9	37.33	1	0.5	0.5
R5	50	1.67	9	35.33	2	1	1
R6	50	1.67	9	31.33	4	2	2

**Table 2:** Conductivity and pH of developed emulsions.

Parameters	Sample Code											
	R1		R2		R3		R4		R5		R6	
Time of Storage	0 h	4 h	0 h	4 h	0 h	4 h	0 h	4 h	0 h	4 h	0 h	4 h
pH	4.81	4.76	4.78	4.80	4.78	4.73	5.23	5.23	5.07	4.94	4.65	4.61
Conductivity (mS/cm)	1.37	1.35	1.28	1.29	1.24	1.22	1.49	1.47	1.35	1.34	1.21	1.21

**Table 3:** The viscosity indices obtained for the synthesized emulsions.

Sample	Viscosity (cP)	Shear Stress (dyne/cm^2^)	SR (s^−1^)	Speed (RPM)	Temperature (°C)
R1	156.74	177.03	186	200	22.31
R2	167.45	230.52	186	200	23.33
R3	220.29	281.03	186	200	22.40
R4	235.81	396.93	186	200	23.19
R5	239.60	435.70	186	200	23.10
R6	241.22	461.80	186	200	22.53

**Table 4:** Physicochemical and mechanical characteristics of the cotton-treated samples (R1–R6) and non-treated fabric (C—control).

Sample	Mass [g/m^2^]	Maximum Force (N)		Elongation to Maximum Force (%)		Permeability to Air (l/m^2^/s)	Water Vapor Permeability (%)	Hydrophilicity (s)
		Warp	Weft	Warp	Weft			
C	168	745	462	12.11	14.52	234.3	34.9	Immediate
R1	176	761	474	14.06	14.71	140.4	36.2	1.20
R2	172	768	468	17.24	21.30	147.2	35.3	1.40
R3	181	763	479	16.80	20.60	146.4	35.3	1.30
R4	182	787	471	15.23	13.95	140.7	29.5	1.40
R5	185	757	463	13.70	15.21	130.4	31.0	1.60
R6	189	755	478	15.81	18.37	129.4	33.1	1.70

**Table 5:** Evaluation of the antibacterial activity exerted by the treated fabrics.

Code	*E. coli*		*S. aureus*	
	Inhibition Zone (mm)	Effect	Inhibition Zone (mm)	Evaluation
R1	29	Good	29	Good
R2	29	Good	29	Good
R3	29	Good	29	Good
R4	29	Good	29	Good
R5	29	Good	29	Good
R6	29	Good	29	Good
C	0	Insufficient	0	Insufficient

## Data Availability

The raw data supporting the conclusions of this article will be made available by the authors upon request.
